# Actionability and precision oncology

**DOI:** 10.18632/oncoscience.236

**Published:** 2015-09-12

**Authors:** Maria Schwaederle, Razelle Kurzrock

**Affiliations:** Center for Personalized Cancer Therapy, Division of Hematology and Oncology, University of California, San Diego, Moores Cancer Center, La Jolla, CA, USA

**Keywords:** actionability, precision medicine, personalized medicine, cancer

Cancer is driven by molecular aberrations that allow oncogenic cells to thrive by growing and eventually metastasizing, and/or by alterations in the tumor or host that are permissive for immune evasion. The development and availability of clinical-grade molecular diagnostic tests that interrogate the genomic portfolio of each patient's tumor, together with the clinical development of a myriad of selective agents that target these alterations or harness the immune system, has enabled precise prosecution of malignancies. This field has been designated “precision” or “personalized” medicine, and it is a combination of both—precise because it is necessary to specifically hone in on the tumor versus normal cells, and personalized because it turns out, based on the complex and distinct biologic/molecular landscape of each person's cancer, that the way to do this differs from individual to individual. The backbone of precision medicine is based on the following precepts: (i) understand the genomic landscape of each tumor and deploy matched targeted therapy; (ii) harness the immune system; (iii) treatment must be tailored to each individual's tumor; (iv) combination regimens are needed to address the complex molecular/biological signature that is the hallmark of many cancers.

Our recent meta-analysis performed on more than 30,000 patients[1] suggests that matched personalized/precision therapy produces by far the best outcome across cancer types and studies, while treating with targeted agents without biomarker selection has the worst success rates, with cytotoxic therapies (which rarely employ biomarkers) being intermediate.

There are however several barriers to a change in paradigm towards a personalized or precision medicine approach. Further, the utility of this approach still remains a matter of debate for several reasons. One oft-repeated misconception is that the majority of tumors do not harbor potentially actionable abnormalities. While this may have been true a few years ago, with the rapid advancement of genomics technology and with multiple new targeted and immunotherapeutic agents now being part of the clinical armamentarium, the rate of actionability has risen. Indeed, in a recent study, we showed that, amongst 439 patients suffering from advanced cancer, 70% had alterations potentially druggable by a Food and Drug Administration (FDA)-approved agent, and 90% of individuals had actionable aberrations if experimental compounds in clinical trials were considered[2]. Indeed, the major hurdle for actionability is now medication access, since protocol eligibility is often strict, sick patients must travel long distances to clinical trials sites, and FDA-approved drugs are expensive with not all payors covering their off-label use[3].

It should however be noted that scientific challenges remain[4]. For instance, some of the most common mutations in cancer involve tumor suppressor genes, and alterations in these genes often confer a worse prognosis[5]. While direct targeting of tumor suppressor genes has proven challenging, indirect targeting by impacting signaling upregulated by tumor suppressors loss may be a viable strategy. For instance, *PTEN* loss results in upregulation of the PI3K/AKT/mTOR pathway, which can then be impacted by cognate inhibitors. Importantly in this regard, the tumor suppressor *TP53* is amongst the most frequently altered gene across tumors, and there are currently no approved agents directly modulating *TP53* function. However, a retrospective study performed in diverse tumor types showed that patients treated with bevacizumab (an anti-angiogenic antibody targeting VEGF-A) and harboring mutant *TP53* had significantly longer progression-free survival than those with wild-type *TP53 (*p<0.001)[6]. The underlying mechanism may relate to the fact that *TP53* mutations are an independent predictor of higher expression of VEGF-A transcripts (P=0.006), with VEGF-A being the direct target of bevacizumab and the ligand for VEGFR1 and VEGFR2[7].

Another pillar of precision medicine is immunotherapy. In this regard, biomarkers may also optimize development of these agents. For instance, PD-L1 expression as well as mismatch repair deficiencies have been associated with high response rates to molecules targeting the PD-1/PD-L1 axis.

A final important conundrum for precision medicine is the complexity and heterogeneity of tumors, even within the same patients. The genomic signature across cancers indicates that, for many patients, there are complicated networks of alterations[8]. As signaling pathways are closely inter-connected and various feedback loops can be activated by the roadblock imposed by a single-agent targeting drug strategy, it seems clear that matched single agent may not satisfy the promises of personalized medicine, and new paradigms for clinical trials are needed. Taken together, technologic advances in molecular diagnostic tests combined with high druggability rates of the detected alterations (informed in part by an optimized understanding of cancer biology) strongly support the deployment of a biomarker-based customized combination treatment strategy.
